# DNA barcoding of *Cloridopsis immaculata*: genetic distance and phylogeny of stomatopods

**DOI:** 10.1080/23802359.2018.1507632

**Published:** 2018-09-10

**Authors:** Shantanu Kundu, Shibananda Rath, Kaomud Tyagi, Rajasree Chakraborty, Avas Pakrashi, Vikas Kumar, Kailash Chandra

**Affiliations:** Centre for DNA Taxonomy, Molecular Systematics Division, Zoological Survey of India, Kolkata, India

**Keywords:** Chilika Lake, mantis shrimps, mtCOI, genetic traits, phylogeny

## Abstract

The changes of coastal topography might have genetically altered the extant species diversity in Chilika Lake. The genetic assessment of stomatopods has never been attempted from this ecosystem. The study generate the first genetic information (mtCOI) of *Cloridopsis immaculata*. DNA sequences of C. *immaculata* shows 12.9% genetic divergence with *Harpiosquilla harpax* and clade as sister species in NJ tree. *Alima, Harpiosquilla*, and *Oratosquilla* shows high congeneric/conspecific genetic divergence (20.9%, 15.7%, and 7.2%) and cladded separately in the phylogeny; correlate to their diverse populations. We recommend more extensive survey of stomatopods and generation of molecular data to resolve the taxonomic uncertainty.

## 1. Introduction

The marine and terrestrial ecosystems are facing a rapid loss of biodiversity in the recent past and that circumstances accelerate to catalogue the extant flora and fauna from natural settings (www.all-species.org, Wilson 2003). The stomatopod crustaceans are one of the charismatic animals in the marine world with high protein content. Thus, the animal has gain huge socioeconomic importance and enormously used as a food source in Southeast Asia and other Mediterranean countries (Barber and Boyce 2006). The taxonomic group comprises over 480 named species (Ahyong 1997, 2004 ). Although, several anecdotal information has been reviewed on this group, but the thorough taxonomic investigation and generation of molecular data are precisely attempted throughout the world (Tang et al. 2010).

Chilika Lake is one of the largest brackish water lagoons in the east coast of India. Due to the connectivity with both freshwater and marine ecosystem, the lake harbors a fraternization of saltwater and freshwater. Thus, the ecosystem allows proliferation of an abundant number of species diversity. Several surveys has been attempted to know the species composition of the lake, and recorded more than 800 vertebrate fauna recorded from this oldest aquatic ecosystem (WWF India 2008). Kemp (1915) reported three stomatopod species; *Cloridopsis immaculata*, *C. scorpio*, and *Oratosquilla interrupta* from Chilika lake. Later on, based on the morphological characters of mouth structure, researchers claimed more inhabiting species and recorded *Harpiosquilla raphidea* from the lake (Ghosh 1995; Rath and Mishra 2013). In the recent past, the Chilika Development Authority (CDA) reported four new records of stomapod species, *Harpiosquilla malagasiensis*, *Harpiosuilla paradipa*, *Harpiosquilla harpax*, and *Squilla harpax* from Chilika Lake and stated total eight extant species. Among all extant stomatopod species, *C. immaculata* is commonly distributed and an economically important fisheries resource of India (Panda et al. 2008).

Nevertheless, due to several natural calamities and anthropogenic threats, an enormous numbers of biota of this lake are now listed in a threatened category (IUCN 2018). Hence, the accurate identification of species, population structure, and the intervention of molecular tools are urgently necessitated for proper conservation management. DNA barcoding is evidenced as a successful supportive tool in systematics research for inventorization of several extant faunal component including stomatopods (Barber and Boyce 2006; Kundu et al. 2018; Laskar et al. 2018). The present study applies combined morphological and DNA barcoding techniques to estimate the stomatopod species diversity in Chilika Lake.

## 2. Materials and methods

### 2.1. Sampling and laboratory analysis

The stomatopod species were collected from Nalabana island of Chilika Lake (19.69 N 85.29 E) in eastern coast of Odisha state. The collected specimens were identified by available keys (Ghosh 1995) and photographed by Nikon D3100 camera. The specimens were preserved in 70% alcohol and deposited in the Crustacea Section of Zoological Survey of India, Kolkata with specific voucher numbers (ZSI SQ1: C-7454/2, ZSI SQ2: C-7455/2, and ZSI SQ3: C-7456/2). The total genomic DNA was extracted following the QIAamp DNA Mini Kit standard protocol (Qiagen, Valencia, CA). The published primer pair, LCO1490: 5′-GGTCAACAAATCATAAAGATATTGG-3′ and HCO2198: 5′-TAAACTTCAGGGTGACCAAAAAATCA-3′ (Folmer et al. 1994) was used for amplification of partial mitochondrial cytochrome c oxidase subunit I (mtCOI) gene segment in a Veriti^®^ Thermal Cycler (Applied Bio systems, Foster City, CA). The 25µl PCR mixture contains 10 pmol of each primer, 100 ng of DNA template, 1X PCR buffer, 1.0–1.5 mM of MgCl2, 0.25 mM of each dNTPs, and 0.25 U of Platinum Taq DNA Polymerase High fidelity (Invitrogen, Life Science Technologies) with the following thermal cycling parameters: 5 min at 94 °C; followed by 40 cycles of 30s at 94 °C, 40s at 49 °C, 1 min at 72 °C, and final extension for 5 min at 72 °C. The PCR amplified products were checked in 1% agarose gel containing ethidium bromide (10 mg/ml). Further, the PCR products were purified using QIAquickR Gel extraction kit (QIAGEN Inc., Germantown, MD), and cycle sequencing products were cleaned by using standard BigDye X Terminator Purification Kit (Applied Biosystems). Sequencing was done bi-directionally in 48 capillary array 3730 DNA Analyzer (Applied Biosystems) following Sanger sequencing methods in the in-house sequencing facilities in the Zoological Survey of India, Kolkata.

### 2.2. Dataset preparation and sequence analysis

The generated sequences were checked by Sequence Analysis software (ABI) and assured by the online BLAST search program and ORF finder. Finally the generated sequences were submitted in the global database (GenBank) to acquire the specific accession number. We screened the GenBank database to acquire the publicly available COI sequences (*n* = 39) of same and related stomatopod species (family Squillidae) and one sequence of Brachypoda species, *Lightiella magdalenina* as an out-group in the dataset. The screened sequences were aligned using ClustalX software (Thompson et al. 1997) and finally, each of the sequences was compared in NCBI through BLASTn and ORF finder to examine the complete alignment and stop codons (http://www.ncbi.nlm.nih.gov/gorf/gorf.html). Primarily, the generated sequences were identified in the online identification system, in GenBank with ‘Highly similar sequences (megablast)’ and BOLD databases with ‘All Barcode Records on BOLD’. The mtCOI sequences were analyzed through Neighbor-Joining (NJ) tree and Kimura 2 parameter (K2P) by using MEGA6 to infer the genetic distance and monophyletic clustering of the studied taxa (Tamura et al. 2013).

## 3. Results and discussion

In this study, we have examined the morphological characters and identified the specimens as *C. mmaculate*. We observed seven specimen with total length varies from 56.3 to 79.6 mm and all the specimen with dactylus of raptorial claw with five teeth, lateral process of 5th thoracic somite broad, lateral process of 6th and 7th thoracic somites posterolaterally rounded. The eyes are small and cornea is bi-lobed, rostral plate as long as broad. Carapace tapered anteriorly, anterolateral spines strong with a rounded ventral lobe, median carina straight. Second abdominal somite is having a dark horizontal line. Abdominal carinae spined, submedian 6, intermediate 6, lateral 5–6. Telson almost as long as broad, three pairs of marginal teeth present; telson denticles: 2, 5–6, 1. Outer margin of uropodal exopod with six movable spines and having a black spot both side. *C. immaculate* is the predominant species in Chilika Lake and commonly distributed in east India. The species further distributed in Pakistan, Arabian coast, Singapore, and Thailand.

**Figure 1.**
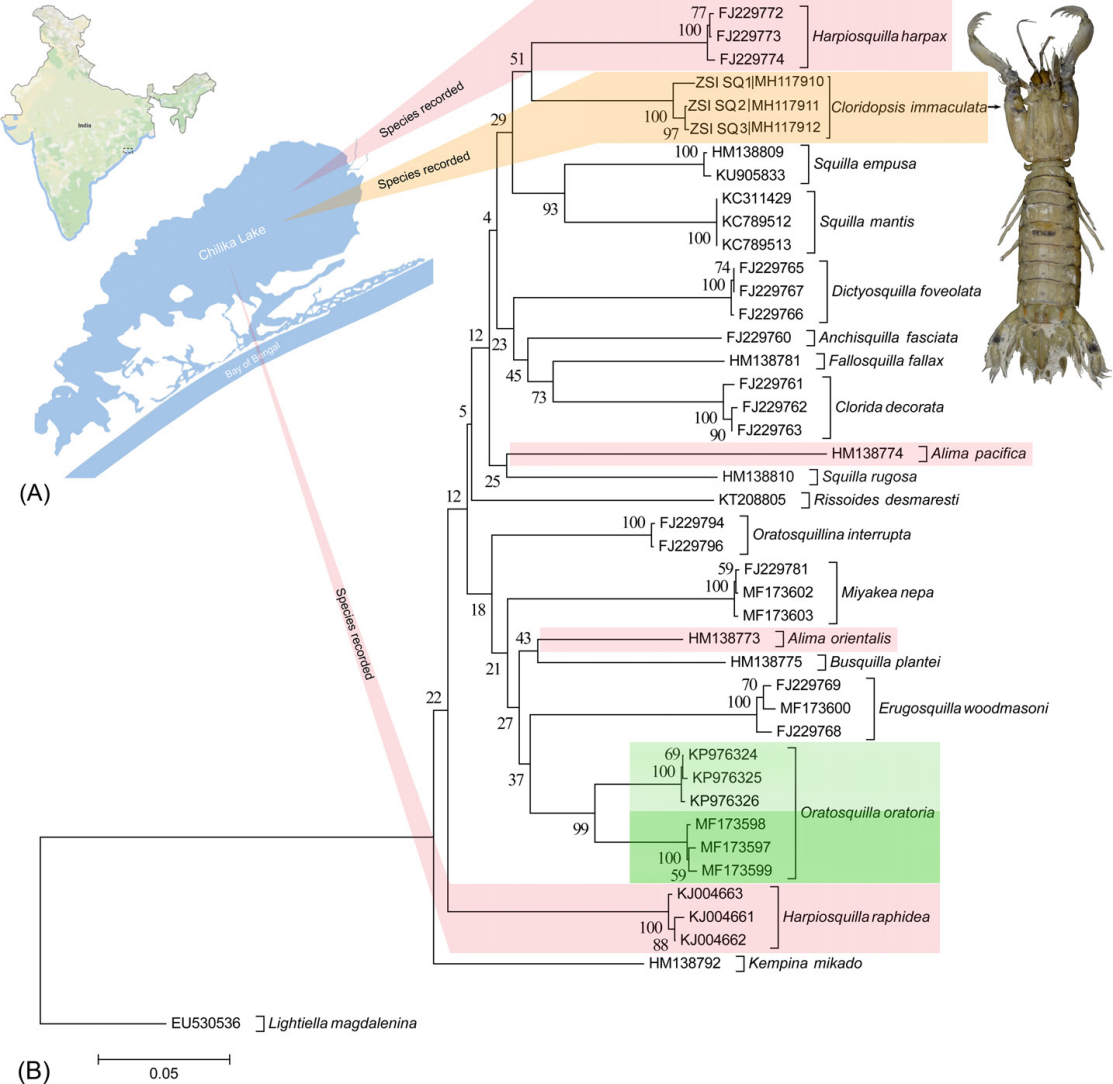
(A) Collection locality of the studied stomatopod species from Chilika Lake of eastern India. (B) Neighbor joining (NJ) tree of the studied stomatopod species with bootstrap support. The Brachypoda species, *L. magdalenina* is used as an out-group in the phylogeny. Green and pink bars represent the ambiguous clade of *Oratosquilla*, *Alima*, and *Harpiosquilla* species in the present dataset correlate to the high genetic variability. Orange bar represents the novel sequences of *C. immaculata* generated in the study. The image of *C. immaculata* photographed by the second author (SR) is superimposed with the tree.

The genetic information of taxonomically identified species is requisite for executing the genetic similarity search in the global database (Moritz and Cicero 2004). Thus, before submitting any novel gene sequences in public platform, it is mandatory to identify the studied specimens. As of now, the genetic information of two known species of genus *Harpiosquilla*, *H. raphidea*, and *H. harpax* are accessible in the GenBank database. The generated sequences of *C. mmaculate* from Chilika Lake were first time annotated (656bp) and submitted into the GenBank database. The generated sequences are shows 88% to 89.03% similarity with *H. harpax* in both GenBank and BOLD database. Due to the lack of publicly available sequences of stomatopod species from diverse geographical regions, the similarity search results are not conclusive to identify the studied species. Further, the estimated NJ tree depicted cohesive clustering of the dataset sequences of specific species with high bootstrap support (Figure 1). Most of the dataset species shows monophyletic clustering except the congeners of *Alima* and *Harpiosquilla*. The overall mean genetic distance of the studied dataset is 18.3%, and within species genetic divergence is range from 0.1% to 1%. The dataset also resulted, 11.3 to 23.9% genetic divergence between the studied species. The generated sequences of *C. mmaculate* shows 12.9% genetic divergence with *H. harpax* and shows sister clade in the dataset. Two sequences of *A. pacifica* and *A. orientalis* shows different clustering with high genetic divergence (20.9%) and in *mtCOI* gene. The database sequences of *H. harpax* and *H. raphidea* also shows distant clustering in the phylogeny with 15.7% genetic divergence. Further, the six database sequences *O. oratoria* shows two distinct clades with high bootstrap support. The two clades of *O. oratoria* shows 7.2% genetic divergence within species, which is comparably high with other studied species. It is evident that, the zoogeography acts as a barrier to gene flow and promoted allopatric diversification in *O. oratoria* species complex (Cheng and Sha 2017). Further, the significant genetic divergence also depicted between the Yellow Sea and East China Sea populations of *Trachypenaeus curvirostris* revealed by the *mtCOI* gene (Han et al. 2015). The Chilika lagoon was connected to the Bay of Bengal during the later stages of the Pleistocene period and due to physical and environmental attributes, it is irregularly connected in recent past (Kundu et al. 2018). The biogeographic process might be altered the genetic structure, and thus the recent attempt is justified to examine DNA data of stomatopod species from Chilika lake to determine the accurate species diversity and phylogenetic relationship.
